# Case report: Local and systemic combination therapy: endoscopic injection of an oncolytic virus with PD-1 inhibitor for an elderly patient with advanced gastrointestinal cancer

**DOI:** 10.3389/fimmu.2025.1719438

**Published:** 2025-11-21

**Authors:** Siqi Luo, Xue Ding, Hong Zhang, Li Dai, Meifeng Zhang, Zhengye Wang, Liangbi Xu, Qian Wang, Xiangren Jin

**Affiliations:** 1 Department of Clinical Medicine, Guizhou Medical University, Guiyang, Guizhou, China; 2 Department of Hepatobiliary Surgery, The Affiliated Hospital of Guizhou Medical University, Guiyang, Guizhou, China; 3 Department of Gastrointestinal Surgery, The Affiliated Hospital of Guizhou Medical University, Guiyang, Guizhou, China; 4 Government Agency Clinic, The Affiliated Hospital of Guizhou Medical University, Guiyang, Guizhou, China; 5 Digestive Endoscopy Center, The Affiliated Hospital of Guizhou Medical University, Guiyang, Guizhou, China

**Keywords:** gastric cancer, colorectal cancer, oncolytic virus, PD-1 inhibitors, elderly patients

## Abstract

Gastric and colorectal cancers present significant therapeutic challenges, particularly in the elderly population, who often have comorbidities and diminished tolerance to standard treatments. This report describes an 85-year-old male with concurrent stage III gastric adenocarcinoma and stage IIIb microsatellite stable colorectal cancer, who declined both surgery and chemotherapy. Subsequently, the patient was treated with an innovative regimen consisting of endoscopic intratumoral injections of Oncolytic adenovirus H101 in combination with the PD-1 inhibitor tislelizumab. Following this combined therapeutic approach, the patient demonstrated notable tumor shrinkage and downstaging, accompanied by a reduction in serum tumor markers, including CEA and CA19-9. Additionally, there was an observed increase in CD8^+^ and CD4^+^ T-cell counts, indicating systemic immune activation. The treatment was well-tolerated, with the only reported adverse event being mild fever. The patient achieved nearly 4 months of progression-free survival and a substantial improvement in quality of life. This case highlights the potential of combining oncolytic virotherapy with PD-1 inhibition as a promising and novel personalized strategy for treating elderly patients with advanced gastrointestinal cancers who are unsuitable candidates for conventional therapies.

## Introduction

With the global aging population steadily increasing, the demand for effective diagnosis and treatment of elderly cancer patients is growing exponentially. The median age of patients diagnosed with colorectal cancer is 66 years, with over 70% of gastric cancer patients aged 60 years or older (1, 2). This demographic shift highlights the importance of adapting cancer treatment protocols to better suit the needs of elderly individuals. However, the aging process is often accompanied by a decline in physiological functions, an increase in comorbidities, and diminished drug resistance, making treatment plans for elderly patients inherently more complex. One of the central challenges in this context is how to effectively balance the therapeutic outcomes with maintaining the quality of life (QoL) for these patients (3, 4).

Currently, the treatment modalities for gastric cancer include surgery, chemotherapy, targeted therapy, immunotherapy, and radiotherapy. However, these traditional approaches often fail to fully meet the needs of elderly patients (5, 6). Many elderly individuals are unable to tolerate the rigors of surgery or chemotherapy due to frailty, poor performance status, or multiple underlying health conditions. Furthermore, these treatments can negatively impact their quality of life, with some patients opting to forgo treatment altogether due to the perceived risks and potential adverse effects. A significant concern when using immune checkpoint inhibitors (ICIs) in elderly patients is the heightened risk of treatment-related adverse events (TRAEs) and the subsequent likelihood of treatment discontinuation (7, 8). These risks are further compounded by the vulnerability of the immune system in aging individuals, which can lead to increased side effects and complications. Therefore, there is a pressing need for more personalized treatment strategies that can reduce the burden of adverse events while still offering effective therapeutic outcomes (9).

Oncolytic virus therapy, a novel and emerging immunotherapy, offers a promising alternative to conventional treatments. This therapeutic approach involves viruses that selectively infect and destroy tumor cells while simultaneously stimulating the immune system to produce an anti-tumor response. Research has demonstrated the efficacy of oncolytic virus therapy in various cancer types. When used in combination with PD-1 inhibitors, this therapy can further enhance immune responses, restore T cell function, and counteract immune suppression within the tumor microenvironment. These combined effects can provide a novel treatment option for elderly cancer patients, who may not tolerate conventional therapies as well (10–12). Oncolytic adenovirus H101 (−20°C, Shanghai Sunway Biotech, Shanghai, China), a recombinant type 5 human adenovirus with a E1B55KD deletion and partial E3 region which can selectively replicate in tumor cells.

This article investigates the application of oncolytic virus endoscopic injection combined with PD-1 inhibitors in the treatment of an elderly patient with gastrointestinal tumors. The patient, who was ineligible for surgery and chemotherapy due to advanced age and cardiovascular disease, experienced significant tumor shrinkage and downstaging through a tailored treatment regimen. This case provides valuable insights into the potential benefits of integrating oncolytic virus therapy with immunotherapy for elderly cancer patients, offering a promising approach for improving treatment outcomes and quality of life for this vulnerable population.

## Case presentation

**Figure 1:**
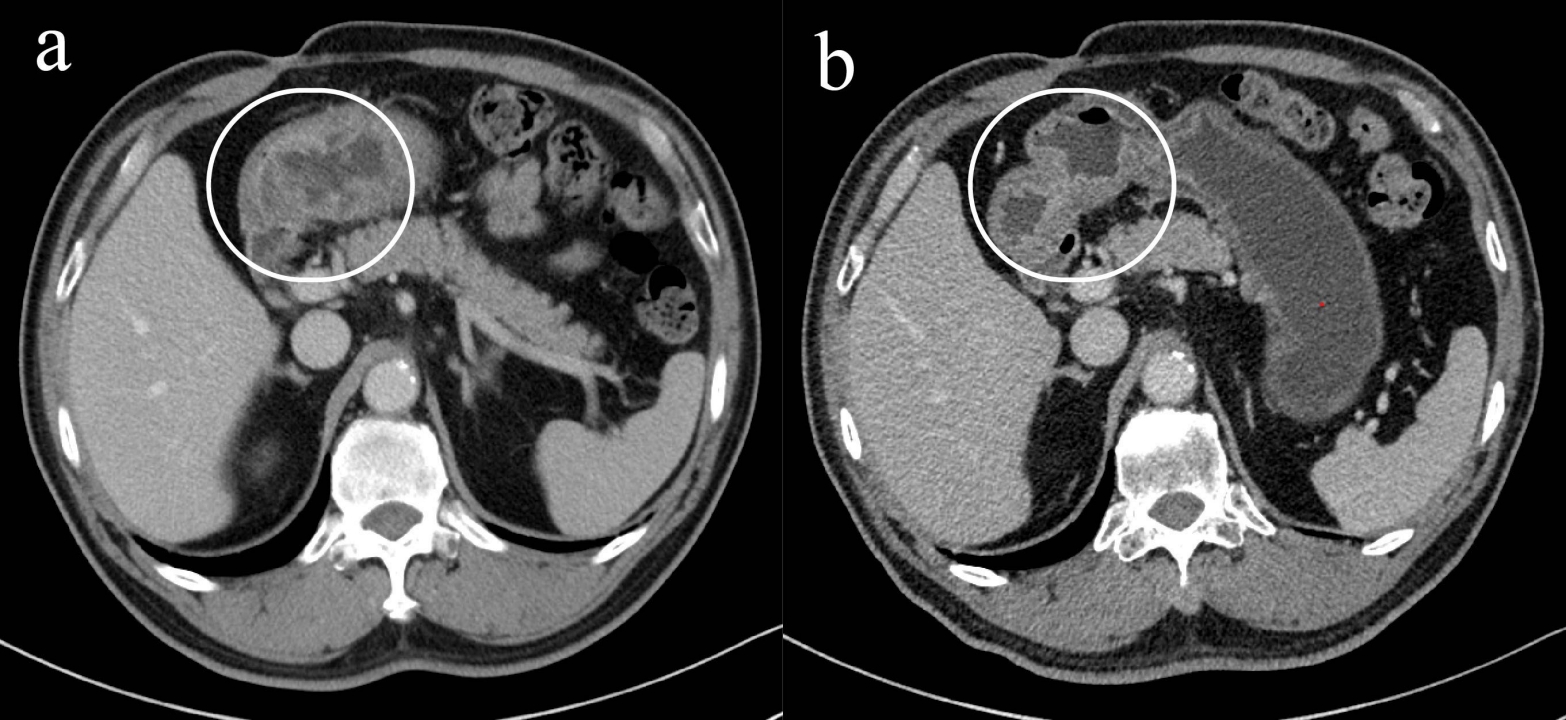
Changes of gastric lesions under gastroscopy, **(a)** On July 30, 2020, **(b)** On October 22, 2020.

**Figure 2:**
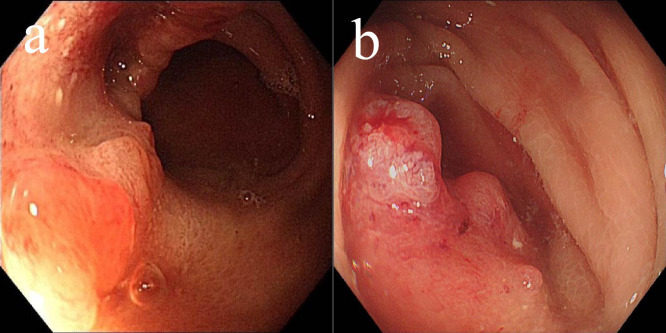
Imaging changes of gastric lesions after treatment, **(a)** On July 26, 2020 (slice thickness: 5mm, arterial phase), **(b)** On October 20, 2020 (slice thickness: 5mm, portal venous phase).

A male patient, aged 85, presented in July 2020 with abdominal pain and bloating after meals. Gastroscopy revealed a large ulcer in the gastric antrum (6×5 cm) (**Figure 1a**), and pathological biopsy confirmed adenocarcinoma. CT scans showed a tumor in the gastric antrum with multiple enlarged lymph nodes in the lesser curvature and surrounding areas. According to the AJCC staging criteria, the clinical stage was cT4aN2M0, stage III (**Figure 2a**). Following a multidisciplinary team (MDT) discussion, a neoadjuvant treatment plan was formulated, involving camrelizumab (IV 200 mg, every 3 weeks), oxaliplatin (IV 130 mg/m², every 3 weeks), and apatinib (oral 250 mg daily for 14 days, every 3 weeks), with the goal of performing surgery after 3 cycles of treatment.

After the second cycle, follow-up gastroscopy revealed a significant reduction in the gastric antrum ulcer. By the third cycle, the ulcer had formed scar tissue (**Figure 1b**). Follow-up CT scans showed significant tumor shrinkage and downstaging, with the clinical stage revised to ycT1bN0M0, stage I, indicating a partial response (**Figure 2b**).

**Figure 3:**
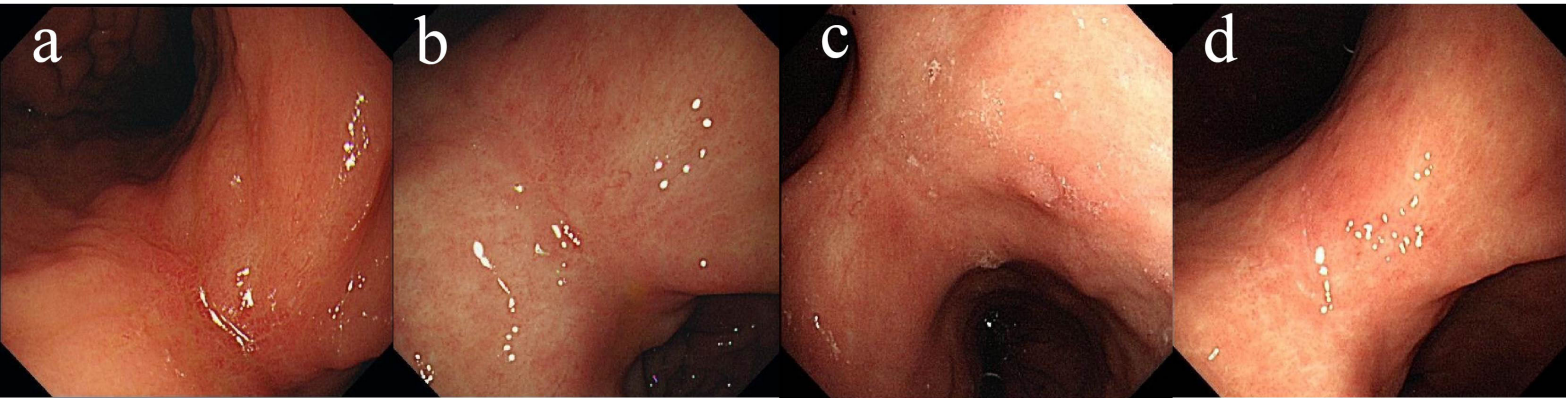
Changes of gastric lesions under gastroscopy, **(a)** On December 25, 2020, **(b)** On April 14, 2021, **(c)** On December 09, 2021, **(d)** On May 02, 2022.

Due to a history of coronary heart disease and the implantation of six coronary stents, the patient considered surgery and endoscopic submucosal dissection (ESD) too risky and refused them. Additionally, the patient declined oral chemotherapy, opting only for regular follow-up. By the end of 2020 and throughout 2022, follow-up gastroscopy showed no significant recurrence of the gastric antrum scar (**Figures 3a–d**). Blood samples were routinely collected for circulating tumor DNA (ctDNA) testing, all of which returned negative results.

**Figure 4:**
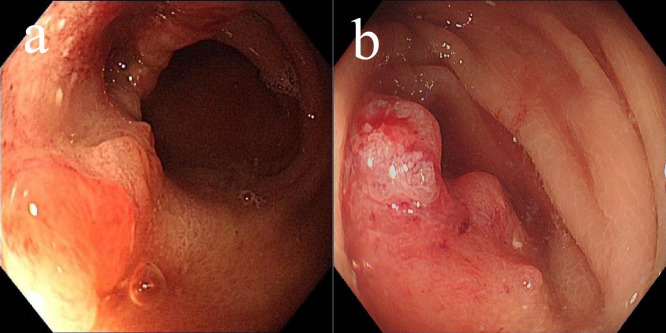
Changes of colonic lesions under colonoscopy, **(a)** On May 02, 2022, **(b)** On May 18, 2025.

In May 2022, the patient presented with difficulty in defecation and abdominal bloating. Colonoscopy revealed an ulcerative neoplasm at the junction of the sigmoid and descending colon (**Figure 4a**), with pathological biopsy confirming adenocarcinoma. According to the AJCC staging criteria, the clinical stage was cT3N1bM0, stage IIIb. Genetic testing revealed wild-type KRAS, NRAS, BRAF, PIK3CA, and no mutations in the UGT1A1 promoter or Exon-1. Microsatellite stability was confirmed (**Table 1**), categorizing the tumor as a “cold tumor.” The patient requested non-surgical treatment and refrained from receiving any further treatment over the subsequent two years.

**Table 1:** Postoperative pathological genetic testing.

Type of testing	Testing result
KRAS	Negative
NRAS	Negative
BRAF	Negative
PIK3CA	Negative
UGT1A1	Negative
Exon-1	Negative
Microsatellite instability	Microsatellite stability

**Figure 5:**
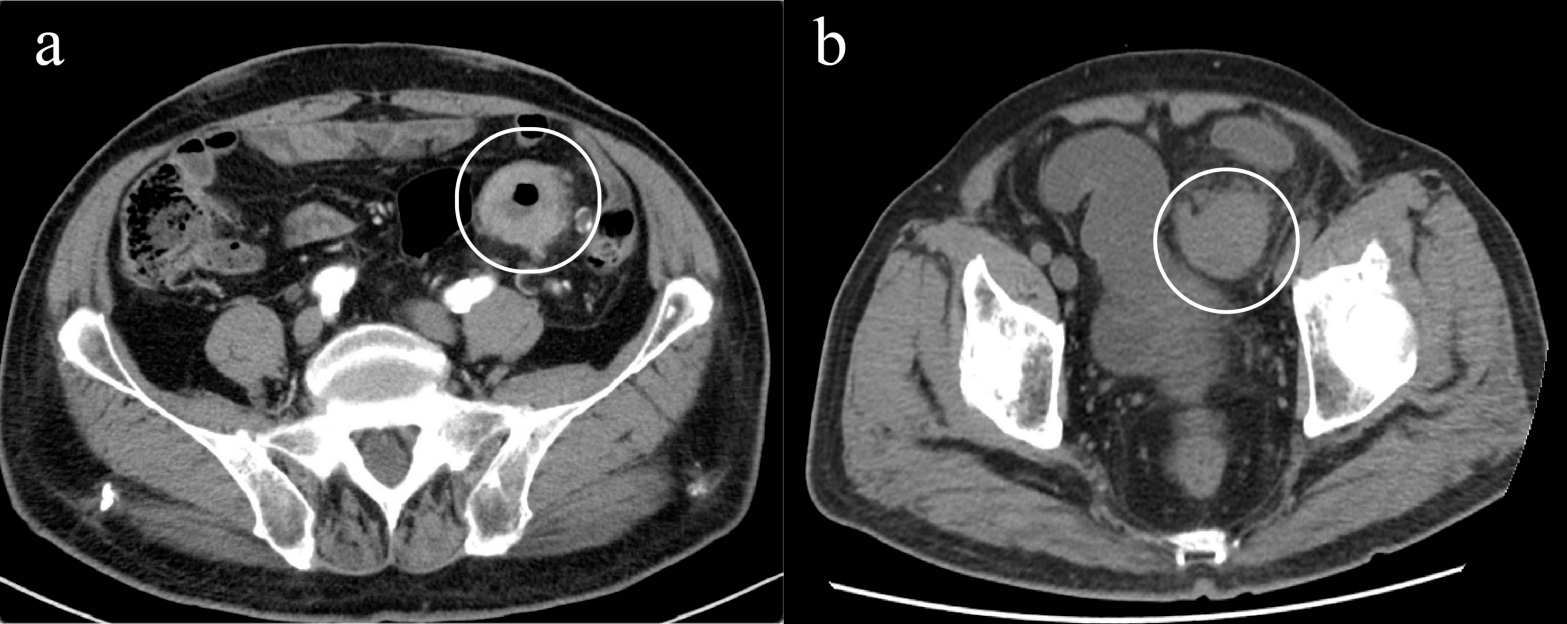
Imaging changes of colonic lesions, **(a)** On May 15, 2025 (slice thickness: 5mm, arterial phase), **(b)** On September 23, 2025 (slice thickness: 5mm, portal venous phase).

In May 2025, due to stenosis of the intestinal lumen, the patient underwent another colonoscopy, which revealed tumor progression (**Figure 4b**). Based on the AJCC staging criteria and a full abdominal CT scan (**Figure 5a**), the clinical stage of colorectal cancer was cT4N2aM0, stage IIIc. The department recommended surgical resection, but the patient again refused due to personal reasons. After reviewing relevant domestic and international research, as well as clinical trial results, and following MDT discussion, a decision was made to proceed with oncolytic virus endoscopic injection combined with immunotherapy. This patient received three intratumoral injections of 1.5 mL Oncolytic adenovirus H101 diluted with 4.5ml normal saline (15.0 × 10^11^ viral particles, 1.05 × 10^11^ PFU, each) on June 4, June 19, and July 3, 2025. Each injection was administered at a dose of 0.3ml on the tumor surface and within the tumor margin area. Each time, a flexible colonoscope (GIF-Q260, 9.2 mm; Olympus, Tokyo, Japan) was inserted at the junction of the sigmoid colon and descending colon, and injection was performed using an endoscopic injection needle (ATE-ZSZ-23×1800×23×5, Jiangsu, China). Immune therapy with tislelizumab (IV 200 mg) was administered the day after each injection.

**Figure 6:**
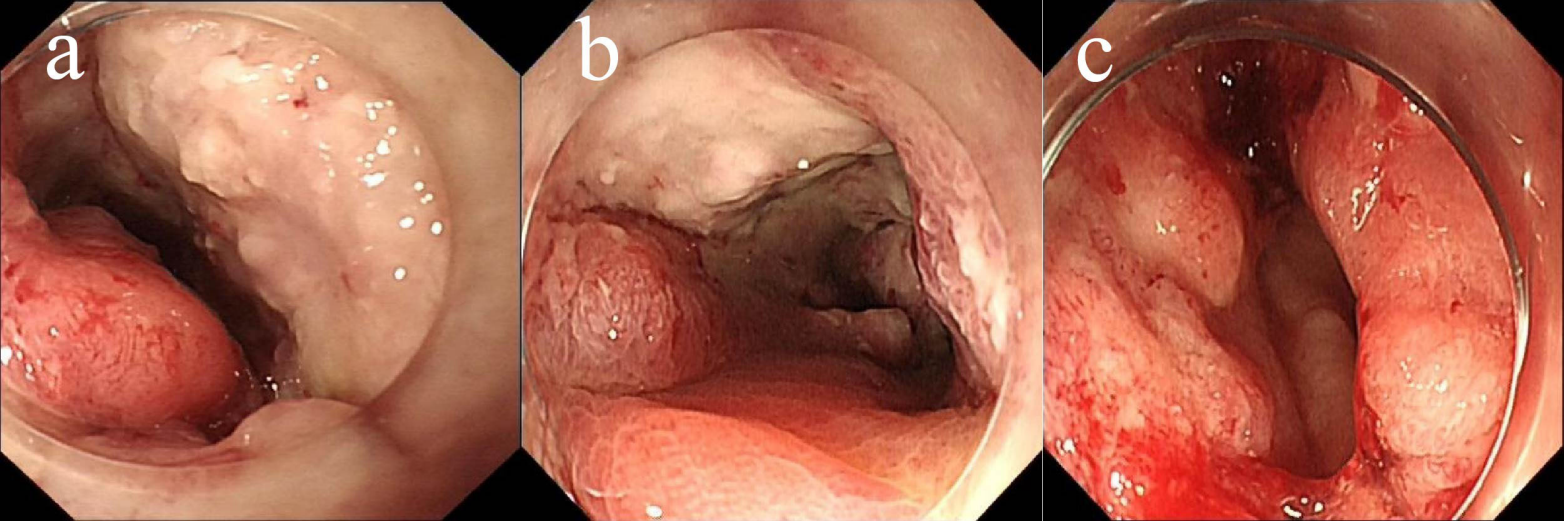
Endoscopic injection of colonic tumors, **(a)** On June 5, 2025, **(b)** On June 19, 2025, **(c)** On July 3, 2025.

**Figure 7:**
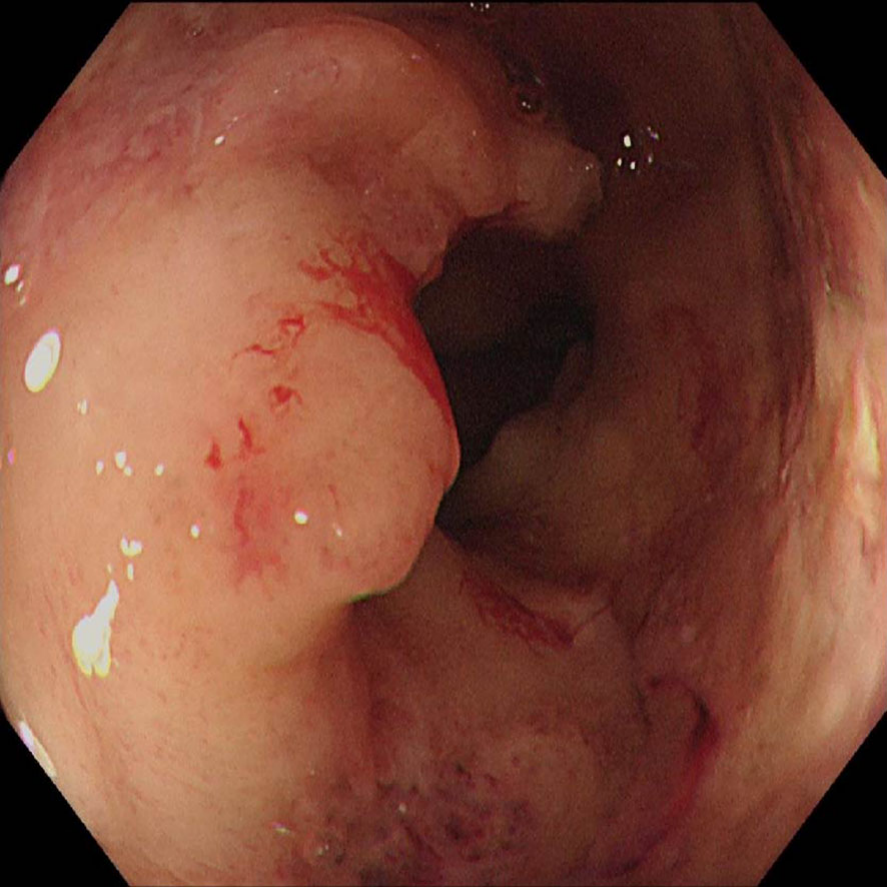
Changes of colonic lesions under colonoscopy, On September 25, 2025.

The first endoscopy revealed the tumor located 50 cm from the anus, with the intratumoral injection completed (**Figure 6a**). The second endoscopy showed significant tumor shrinkage, and the endoscope could barely pass through the narrowed area (**Figure 6b**). After the third treatment, the endoscope passed smoothly, and the tumor had further shrunk (**Figure 6c**). A repeat colonoscopy, performed in September 2025, revealed no enlargement of the lesion or active bleeding (**Figure 7**). Based on the AJCC staging criteria and a full abdominal CT scan (**Figure 5b**), the clinical stage of colorectal cancer was revised to cT3N1aM0, stage IIIb.

**Figure 8:**
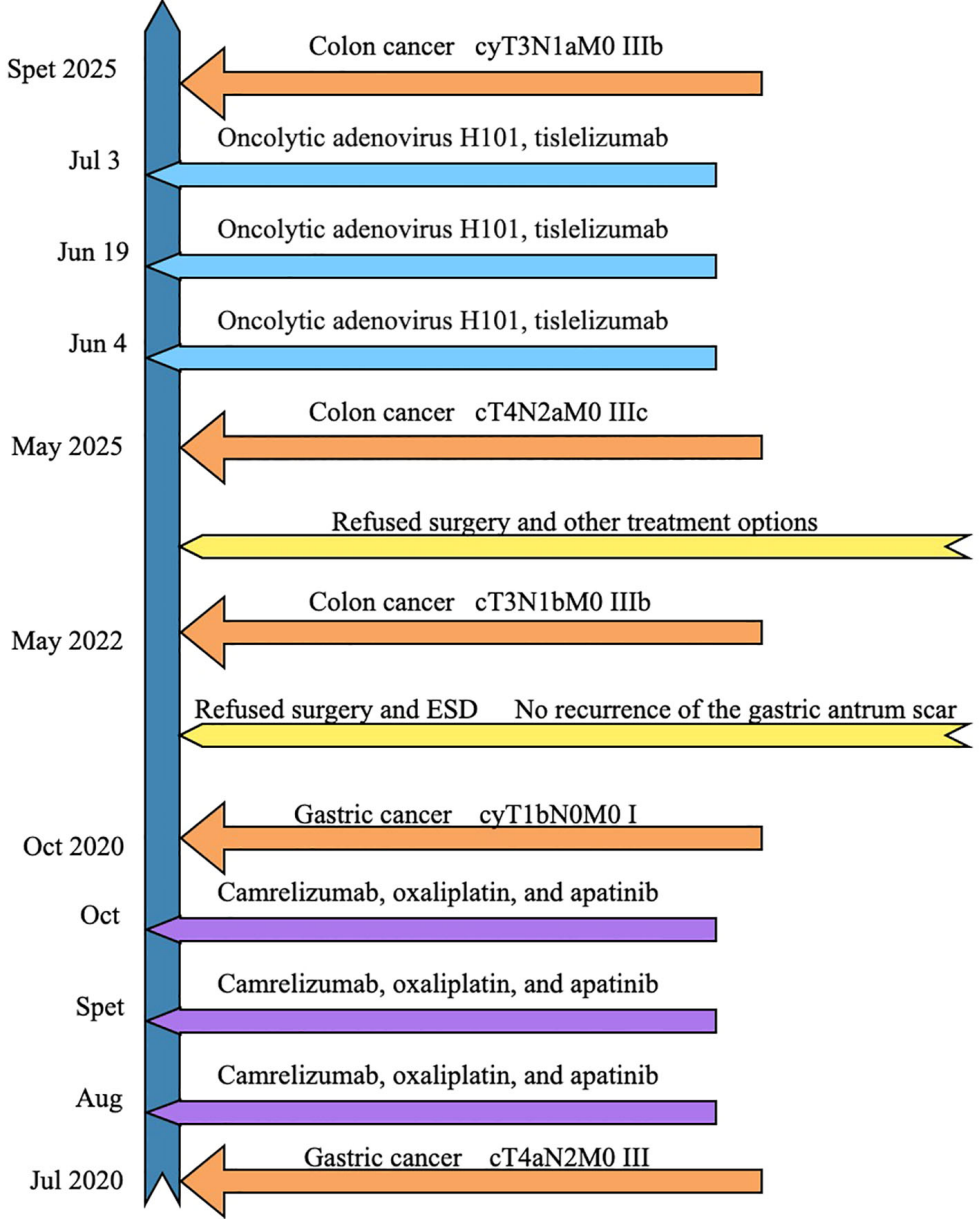
Treatment process.

Currently, the patient is eating normally and has been living without progression for nearly four months (**Figure 8**).

**Figure 9:**
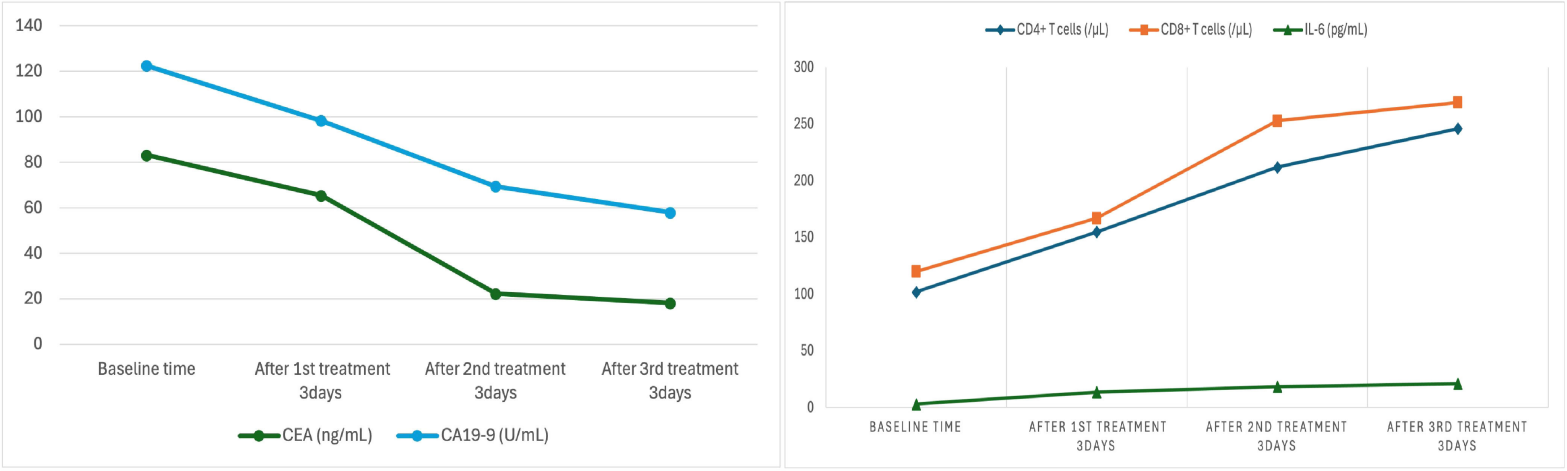
Changes in CEA, CA19-9, CD4^+^ T cells, CD8^+^ T cells and IL-6 during treatment.

During treatment, the patient’s serum tumor markers significantly decreased: CEA decreased from 83.20 ng/mL to 18.2 ng/mL, and CA19–9 decreased from 122.62 U/mL to 58 U/mL. Immunological monitoring showed a significant increase in CD8^+^ T cells, from 102/μL to 246/μL, CD4^++^ T cells increased from 120/μL to 269/μL and interleukin-6 increased from 3.2 pg/mL to 21.2 pg/mL (**Table 2**) (**Figure 9**).

**Table 2:** Laboratory test results before and after treatment.

Time laboratory tests	Baseline time	After 1^st^ treatment 3days	After 2^nd^ treatment 3days	After 3^rd^ treatment 3days
CEA (ng/mL)	83.2	65.4	22.3	18.2
CA19-9 (U/mL)	122.52	98.3	69.4	58
CD4+ T cells (/μL)	102	155	212	246
CD8+ T cells (/μL)	120	167	253	269
IL-6 (pg/mL)	3.2	13.7	18.5	21.2

Regarding adverse reactions, no thrombocytopenia was observed during the three treatments. No fever occurred after the first treatment; however, the second treatment resulted in a rise in temperature (38.5°C), which subsided with symptomatic treatment. The third treatment caused a fever of 39°C accompanied by chills, which returned to normal after medication. Additionally, quality of life was evaluated during all three treatments using the EORTC QLQ-C30 scale (**Table 3**). Results showed improvements in six functional domains: physical, role, emotional, cognitive, social functions, and overall health, compared to pre-treatment levels. Fatigue, nausea and vomiting, pain, difficulty breathing, insomnia, loss of appetite, constipation, and diarrhea showed a decreasing or stable trend.

**Table 3:** EORTC QLQ-C30 scale score before and after treatment.

Time index	Baseline time	Scores after 1^st^ treatment	Scores after 2^nd^ treatment	Scores after 3^rd^ treatment
Physical function	68	75.3	78.2	85.6
Role function	66.7	69.2	75	79.3
Emotional function	45.8	58.2	60.5	82
Cognitive function	69.2	75.2	77.4	78.5
Social function	58	62.5	79.2	80.2
Overall health condition	41.7	57	73	79.2
Fatigue	69.2	55	23.4	8
Nausea and vomiting	58.5	38.7	22.3	0
Pain	78.1	50.2	33.8	3.4
Difficulty breathing	33.3	28.3	10	0
Insomnia	88	62.4	37.2	12
Loss of appetite	98	60.2	25.6	8.4
Constipation	92.3	55.3	31.3	7
Diarrhea	0	0	0	0
Economic hardship	0	0	0	0

In conclusion, this case demonstrates that oncolytic virus endoscopic injection combined with immunotherapy can achieve significant therapeutic effects in an elderly patient with heterogeneous gastrointestinal malignant tumors. The treatment was associated with minimal adverse reactions and controllable safety, presenting a promising option for patients unable or unwilling to undergo surgery.

## Discussion

This case report presents an 85-year-old male patient diagnosed with gastric antrum adenocarcinoma and heterogeneous colorectal cancer. Following the diagnosis of advanced colorectal cancer, the patient declined surgery and traditional chemotherapy due to advanced age and comorbidities. In response, the treatment team implemented an innovative, personalized approach: endoscopic injection of Oncolytic adenovirus H101 combined with intravenous injection of tislelizumab. This combination therapy significantly reduced tumor burden, improved the patient’s quality of life, and was associated with manageable adverse effects.

Oncolytic viruses selectively infect and destroy tumor cells while simultaneously activating immune responses that enhance anti-tumor effects. Genetically modified oncolytic viruses replicate selectively within tumor cells, releasing tumor-associated antigens (TAAs) and danger-associated molecular patterns (DAMPs) and pathogen-associated molecular patterns (PAMPs). These signals trigger T-cell-mediated immune responses (7). In this case, following treatment, there was a notable increase in the patient’s CD8^+^ and CD4^+^ T cell counts, indicating successful immune activation.

Gastrointestinal tumors typically present with an immune-suppressive tumor microenvironment (TME). However, oncolytic viruses can facilitate the polarization of tumor-associated macrophages and enhance CD8^+^ T cell infiltration, thus reversing the “cold” tumor microenvironment into a more immune-reactive “hot” state (13). Specifically, in colorectal cancer research, oncolytic viruses have shown promise in transforming immune-cold tumors into immune-hot tumors, thereby enhancing the efficacy of immunotherapies (14).

In recent years, the combination of oncolytic viruses and immune checkpoint inhibitors (ICIs) has garnered significant research attention. Oncolytic viruses promote T-cell infiltration into tumor tissues through the induction of tumor cell death and TME remodeling. PD-1 inhibitors can relieve T-cell functional suppression, further boosting immune responses. This “activation + suppression relief” model has demonstrated promising results in clinical studies, such as those involving liver metastatic colorectal cancer, where oncolytic viruses combined with localized chemotherapy successfully induced anti-tumor immune responses (15).

The NCT04755543 study indicated that the combination treatment exhibited good safety, with mild fever and injection site pain being the primary adverse reactions. No severe adverse events were observed. In terms of efficacy, the objective response rate was 35.9%, and partial responders experienced remission lasting up to 313 days, far surpassing the outcomes of traditional treatments (16).

Based on the viral replication dynamics, immune response activation time, and clinical feasibility of H101, a dosing interval of Day 0, Day 15, and Day 30 was chosen (25, 26). The initial injection on Day 0 initiates viral replication and triggers the early immune response, laying the foundation for subsequent immune activation. The second injection on Day 15 coincides with the peak of the immune response, further enhancing T cell activation and memory response. The third injection on Day 30 aims to maintain sustained immune pressure and prevent tumor immune escape. This regimen design references the clinical protocol and safety data from Zhang et al. on oncolytic virus therapy for malignant ascites, aiming to balance viral clearance with immune stimulation, while minimizing cumulative toxicity and ensuring adequate immune response development (24).

For elderly patients with comorbidities, treatment safety is of paramount importance. In the context of colorectal cancer treatment, research has shown that oncolytic virus M1 exerts strong oncolytic effects without inducing serious systemic toxicity (17). One study by Zhang demonstrated that oHSV2 treatment in a mouse colorectal cancer model did not cause weight loss, and no necrosis or ulcers were observed at the injection sites (18). This research provides a foundation for the clinical application of oncolytic viruses. Similarly, Emma’s study found no grade 3 or higher treatment-related adverse events in patients with liver metastatic colorectal cancer who received hepatic artery infusion of oncolytic virus TG6002 combined with oral 5-fluorocytosine (19). In this case, fever was quickly alleviated with symptomatic treatment, confirming the manageable nature of such reactions.

During the course of combination therapy with an oncolytic virus and a PD-1 inhibitor, the patient developed a transient febrile episode. Serial immunological monitoring revealed a marked post-treatment increase in peripheral CD4^+^, CD8^+^ T-cell counts and IL-6, showing a clear temporal correlation with the onset of fever. Approximately three days after the second treatment cycle, both CD4^+^, CD8^+^ T-cell and IL-6 counts peaked, coinciding precisely with the development of fever. This time-dependent relationship suggests that the febrile response was most likely driven by treatment-induced immune activation rather than by infectious causes.

Consistent with this observation, previous clinical studies involving oncolytic virus–based immunotherapy, such as talimogene laherparepvec (T-VEC), have identified fever as one of the most common adverse events, occurring in nearly 47% of treated patients (22). The underlying pathophysiology is thought to involve immune system hyperactivation and subsequent cytokine release syndrome (CRS). Activation of immune effector cells leads to the release of proinflammatory cytokines—including (IL-6), tumor necrosis factor-α (TNF-α), and interferon-γ (IFN-γ)—which collectively mediate systemic inflammatory responses characterized by fever, chills, and hypotension (23).

In the present case, the fever occurred early during combination therapy and was temporally associated with a rapid rise in peripheral T-lymphocyte counts. The absence of clinical or microbiological evidence of infection further supports an immune-mediated etiology. This pattern indicates robust activation of the antitumor immune response, suggesting that the oncolytic virus and PD-1 inhibitor may have exerted synergistic effects in stimulating host immunity. Nevertheless, excessive immune activation carries a potential risk of systemic inflammatory complications. Clinicians should therefore maintain close surveillance for immune-related adverse events (irAEs), particularly cytokine-mediated inflammatory responses, and initiate appropriate supportive or immunomodulatory measures when necessary to ensure treatment safety.

Taken together, the transient fever observed in this patient during oncolytic virus and PD-1 inhibitor combination therapy most likely represents an immune activation–related inflammatory response rather than an infection. This phenomenon reflects effective immune engagement and antitumor activation induced by the combined regimen. However, it also underscores the need for vigilant monitoring, early differentiation of immune-mediated versus infectious causes, and timely clinical intervention to balance therapeutic efficacy with immune-related toxicity management.

Safety has also been enhanced by altering the administration route. Local delivery via intratumoral or endoscopic injection significantly reduces the risk of systemic exposure. Several clinical trials in Japan, including those with HF10 and OBP-301 endoscopic injections, have confirmed the safety of this approach, exemplifying the benefits of local precision delivery (20, 21). This case highlights the successful application of the local delivery strategy, effectively ensuring patient safety.

It is important to acknowledge the limitations of this case report. This report is intended primarily as a means of sharing clinical experience and facilitating academic exchange. It serves to illustrate the potential of oncolytic virus endoscopic injection combined with PD-1 inhibitors as a novel and feasible therapeutic option for elderly patients who are ineligible for conventional treatments. The promising outcomes observed in this case warrant further validation through more rigorous research.

Moving forward, we plan to initiate broader, multi-center clinical investigations, including both single-arm and randomized controlled trials, to systematically evaluate the efficacy and safety of H101 in combination with ICIs across various cancer types.

This case provides a novel treatment strategy for elderly patients with gastrointestinal tumors. The combination of oncolytic viruses and immune checkpoint inhibitors offers a new perspective on the personalized treatment of elderly cancer patients, establishing a foundation for future research and clinical practice in this field.

## Data Availability

The original contributions presented in the study are included in the article/supplementary material. Further inquiries can be directed to the corresponding author.
